# A Case of Traumatic Tetanus, and Recovery

**Published:** 1851-02

**Authors:** Theodore S. Bell

**Affiliations:** Louisville, Ky.


### Art. IV.—*A Case of Traumatic Tetanus, and recovery.* By Theodore S. Bell, M.D., of Louisville, Ky.

A young man, named John Varali, aged 22 years, called at my office in the latter part of October, on account of a severe wound of the hand, which had been inflicted with a circular saw. The thumb was severely cut; the first bone being crushed into fine spiculæ, and two of the fingers were injured in a less degree. Feeling anxious to save the injured members, I dressed each one with the roller bandage. The hope of success did not rest upon a very firm foundation, for the young man had been unhealthy from infancy. He has for many years been subject to a singular species of chorea. But the wounds referred to, seemed to do very well for some days, and the treatment, by the bandage, was continued for two weeks. About the end of that time, the thumb was threatened with gangrene, and, in spite of quinine, wine, and nutritious diet, the end of the thumb was destroyed by mortification. But at the point of separation healthy pus was found, and the condition of the granulations was promising. Fomentations were constantly applied to the thumb for several days, but trismus manifested itself in the third week of the wound. The fingers were entirely healed at the time the trismus commenced. The trismus was accompanied by all the symptoms that are usually present in such cases. The jaws were rigid, the tongue stiff, the deglutition of liquid, even, was a matter of great difficulty, and there was stricture of the chest.

After the spasmodic condition of the jaw was well established, emprosthotonos and opisthotonos manifested their presence by unmistakeable signs, and in a considerable experience with tetanus, I have never seen those convulsive actions as constant and severe in any other case. There were times when the face of the patient was drawn nearly to his toes; at other times the back of the head was thrown nearly to the heels. The convulsive action was so great that a table standing near the patient was often thrown across the room, by the sudden accession of a spasm. The patient sometimes sat in a large rocking chair, and was held in it during the spasm; at other times he sat on a lounge, near a partition wall, and the opisthotonos was so violent that a hole was broken through the partition, on one occasion, by the backward movement of the head. The sufferings of the patient were beyond anything I have ever witnessed. He had no rest day or night, and for weeks, neither the trismus, the opisthotonos, nor emprosthotonos seemed to yield in any degree.

In the early part of the tetanic features, my own sufferings with a carbuncle on the left hand were so great, that I was compelled to request Professor J. B. Flint to take charge of Varali for me. He very properly ordered a solution of the extract of the Canabis Indica, in doses of 2½ grains of the extract, every two or three hours. Dr. Flint was impressed with the belief that this extract was of some service, and when I resumed the treatment of the case, I continued it. But for a considerable length of time I saw but little if any mitigation. The spasms were as frequent, and seemed to be as violent as at any time of their career, while under my observation. Partial ease was obtained for a short time, immediately after the action of purgatives. The constipation was unusually great, even for a case of tetanus. On this account, I abandoned all the ordinary preparations of opium, and resorted to McMann's elixir. At the same time I doubled the dose of the Canabis Indica. By purging the patient freely, in the afternoon of almost every day, and the use of the elixir at night, I was able to procure him some sleep, and he often slept several hours at a time. The spasms were less frequent, but were not mitigated in their violence, nor did the trismus abate in any degree. The emprosthotonos was the first variety of the spasms that disappeared, and at the end of four weeks from the commencement of the tetanic attack, the trismus and opisthotonos were considerably mitigated, and finally disappeared.

Throughout the whole of this attack the patient was actively treated. The wound was regularly dressed with warm poultices, and sometimes with the addition of laudanum to the poultice. The patient was a member of the Washington Fire Company, and was, of course, well nursed. His family were unremitting in their attentions. All that could be done by medicine, was done, all that faithful and judicious nursing could do, was freely rendered. The strength was supported by nourishment adapted to the case, and wine of a superior quality formed a leading element of the nourishment. In the repeated efforts to give the sufferer sleep by the use of narcotics, the constipation that naturally belongs to tetanus was increased, and all the ordinary preparations of opium were abandoned on this account, and with the less regret, from the fact that they seemed to render no service to the case. I am not prepared to say that much was done, in controlling the tetanus, by the Canabis Indica, while in my charge, though I faithfully used it up to the termination of the case. There were times in which it was not given for 12 or 16 hours, and the evils were not increased by the omission. But a neglect of purgatives invariably aggravated the spasms, and their action was always followed by decided mitigation for irregular intervals of time. The Elixir of Opium also, usually gave ease, and was generally followed by some sleep.

Throughout the whole of this tetanic attack, the wound of the thumb remained stationary. It seemed to grow, neither better nor worse, during the existence of tetanus through its term of four weeks, and I continued to dress the wound, for more than a month after the tetanus had disappeared. The thumb is now well, with the loss of the first joint.

I have called this a case of tetanus, because all the symptoms of that malady were present in a marked degree. But it is possible that the attack may have been an aggravation of the long standing chorea, and may have simulated tetanus, or the chorea may have exerted some influence on the tetanus. The trismus came on more sluggishly than is usual, but I have seen considerable varieties in this feature. I have seen it come in all its fulness at the very onset of its appearance, and I have seen it develop itself gradually, but never so slowly as in the young man whose case is under consideration. The recovery of this patient surprised me very much, as it did every body who knew any thing of the case.

In presenting a portraiture of this remarkable case, it is proper to mention that some five or six years since, I attended young Varali in a case something like the one recorded above. He was suffering then from a wound, and had well marked tetanic symptoms, from which he recovered much sooner, than from the recent attack. I entertained no doubt about the tetanic character of the first attack, nor do I know that there is much reason for a doubt about the second.

It is gratifying to add, that the health of the subject of these remarks is much better since his recent recovery, than I have known it to be for ten years.

